# Supplementary material for: Exposure to asylum seekers and changing support for the radical right: A natural experiment in the Netherlands
Source: PLoS One. 2021 Feb 2;16(2):e0245644. doi: 10.1371/journal.pone.0245644 (PMC7853446; doi:10.1371/journal.pone.0245644)
Supplement: S1 Appendix — (DOCX) [file pone.0245644.s002.docx]

**Appendix**

| **Appendix A1.** Predicting participation in Wave 2. (N_i_ = 26.064). | | | |
| --- | --- | --- | --- |
|  | Model 1 | Model 2 | Model 3 |
| Intercept | 1.160* | 1.194* | 1.165* |
|  | 0.016 | 0.015 | 0.016 |
| PVV support wave 1 | 0.201* |  | 0.188* |
|  | 0.041 |  | 0.043 |
| Exposure to asylum seekers in regular ASC |  | 0.000 | 0.000 |
|  |  | 0.001 | 0.001 |
| Exposure to asylum seekers in temporary ASC |  | -0.001 | -0.001 |
|  |  | 0.001 | 0.001 |
| Exposure to asylum seekers in crisis ASC |  | 0.002 | 0.001 |
|  |  | 0.003 | 0.003 |
| Exposure to asylum seekers in regular ASC  * PVV support |  |  | -0.001 |
|  |  |  | 0.009 |
| Exposure to asylum seekers in temporary ASC * PVV support |  |  | 0.001 |
|  |  |  | 0.004 |
| Exposure to asylum seekers in crisis ASC  * PVV support |  |  | 0.008 |
|  |  |  | 0.009 |
|  |  |  |  |
| Log likelihood | -14141.17 | -13775.1 | -13763.7 |
| * p<0.05, + p<0.10; (two-tailed test). |  |  |  |
| *Sources: 1VOP, COA.* |  |  |  |

| **Table A2.** Multinomial fixed effects models predicting voting intention for the PVV (standard errors in parentheses; N_i_ = 4,233; N_nb_ = 1,928). | | | | | | | |
| --- | --- | --- | --- | --- | --- | --- | --- |
|  | Right-wing  vs.  PVV | Anti-establishment  vs.  PVV | | Demobilized  vs.  PVV | | Other parties  vs.  PVV | |
| Wave 2 (Ref: wave 1) | -0.910* | -2.008* | -0.826* | | -0.816* | |  |
|  | (0.093) | (0.105) | (0.083) | | (0.083) | |  |
| Exposure to asylum seekers in regular ASC | -0.004 | -0.005 | -0.007 | | -0.018 | |  |
|  | (0.016) | (0.016) | (0.016) | | (0.017) | |  |
| Exposure to asylum seekers in temporary ASC | -0.080 | -0.079 | -0.064 | | -0.065 | |  |
|  | (0.077) | (0.073) | (0.071) | | (0.071) | |  |
| Exposure to asylum seekers in crisis ASC | -0.037+ | -0.027 | -0.022 | | -0.034+ | |  |
|  | (0.020) | (0.019) | (0.019) | | (0.019) | |  |
| Threat | -0.207* | -0.365* | -0.191* | | -0.324* | |  |
|  | (0.073) | (0.088) | (0.066) | | (0.066) | |  |
| Contact non-western | 0.003 | -0.062 | -0.069* | | -0.056+ | |  |
|  | (0.038) | (0.045) | (0.033) | | (0.034) | |  |
| Log likelihood | -2,545 | | | | | |  |

*Sources: 1VOP, COA.*

+ p<0.10; * p<0.05; (two-tailed test).

Notes: Right wing: VVD, CDA and SGP; Anti-establishment: SP; Other parties: PVDA, D66, CU, GL, PvdD, 50PLUS and ‘another party’; Demobilized ‘I don’t know’, ‘blank vote’ and ‘I’m allowed to vote, but I wouldn’t’. To create this categorization, we used information from the 2014 Chapel Hill Expert Survey on party position on immigration policy as well as the salience of anti-establishment and anti-elite rhetoric [4].

| **Table A3.** Robustness checks. Fixed effects models predicting voting intention for the PVV (standard errors in parentheses). | | | | | | | | | | | | |
| --- | --- | --- | --- | --- | --- | --- | --- | --- | --- | --- | --- | --- |
|  | Original estimates | | time heterogeneity in exposure to asylum seekers in crisis ASC | | controlling for days crisis ASC were used | | binarization of exposure measures | | weighted sample | | matched sample | |
| Wave 2 (wave 1 = ref.) | 1.084 | * | 1.100 | * | 1.099 | * | 1.112 | * | 1.157 | * | 0.960 | * |
|  | (0.065) |  | (0.065) |  | (0.065) |  | (0.066) |  | (0.101) |  | (0.191) |  |
| Exposure to asylum seekers in regular ASC | 0.008 |  | 0.008 |  | 0.010 |  | -0.415 |  | 0.037 |  | 0.004 |  |
|  | (0.014) |  | (0.014) |  | (0.016) |  | (0.512) |  | (0.053) |  | (0.009) |  |
| Exposure to asylum seekers in temporary ASC | 0.061 |  | 0.060) |  | 0.060 |  | 0.503 |  | 0.152 | + | 0.066 |  |
|  | (0.067) |  | (0.067) |  | (0.066) |  | (0.642) |  | (0.086) |  | (0.070) |  |
| Exposure to asylum seekers in crisis ASC | 0.029 | + | 0.002 |  | 0.064 | * | 0.120 |  | 0.037 | + | 0.035 | + |
|  | (0.017) |  | (0.002) |  | (0.025) |  | (0.286) |  | (0.020) |  | (0.020) |  |
| Days crisis ASC were used |  |  |  |  | -0.113 | * |  |  |  |  |  |  |
|  |  |  |  |  | (0.043) |  |  |  |  |  |  |  |
| Threat | 0.265 | * | 0.265 | * | 0.264 | * | 0.267 | * | 0.421 | * | 0.385 | + |
|  | (0.059) |  | (0.058) |  | (0.059) |  | (0.058) |  | (0.086) |  | (0.151) |  |
| Contact non-western | 0.034 |  | 0.033 |  | 0.029 |  | 0.033 |  | 0.095 | * | -0.031 |  |
|  | (0.029) |  | (0.029) |  | (0.030) |  | (0.029) |  | (0.048) |  | (0.082) |  |

*Sources: 1VOP, COA.*

+ p<0.10; * p<0.05; (two-tailed test).

| **Table A4**. Weighing statistics | | | | | |
| --- | --- | --- | --- | --- | --- |
|  | Target | unweighted | | weighted | |
|  | % | N | % | N | % |
| women | 0.50 | 279.00 | 0.20 | 690.21 | 0.50 |
| men | 0.50 | 1110.00 | 0.80 | 698.79 | 0.50 |
|  |  |  |  |  |  |
| age1: 18-35 | 0.24 | 39.00 | 0.03 | 117.00 | 0.08 |
| age2: 36-55 | 0.34 | 266.00 | 0.19 | 574.18 | 0.41 |
| age3: 55+ | 0.42 | 1084.00 | 0.78 | 697.82 | 0.50 |
|  |  |  |  |  |  |
| educ1: low | 0.32 | 300.00 | 0.22 | 524.70 | 0.38 |
| educ2: medium | 0.39 | 520.00 | 0.37 | 577.03 | 0.42 |
| educ3: high | 0.28 | 569.00 | 0.41 | 287.27 | 0.21 |

Notes: Population targets for 2015 obtained from Statistics Netherlands.

| **Table A5.** Balance statistics | | | |
| --- | --- | --- | --- |
|  | means | | |
|  | Treated  (N=111) | Control all (N=1,274) | Control matched (N=111) |
| Male (female=ref.) | 0.784 | 0.800 | 0.730 |
| Age^a^ | 0.108 | -0.009 | 0.111 |
| Education^a^ | 0.063 | -0.006 | 0.217 |
| Contact non-western^a^ | 0.064 | -0.006 | 0.014 |
| Threat^a^ | -0.060 | 0.005 | 0.001 |
| Proportion non-western minorities neighbourhood^a^ | -0.024 | 0.002 | 0.013 |
| Economic deprivation neighbourhood^b^ | 0.181 | -0.016 | 0.132 |

Notes: ^a^ Variables are Z-standardized before matching procedure; ^b^ variable has been Z-standardized after a log-transformation

| **Table A6**. Difference in Differences estimator of increased exposure to asylum seekers (i.e. treatment) on support for PVV, via a linear probability model. | | | | | |
| --- | --- | --- | --- | --- | --- |
| Model specification | | | DiD estimator | | |
| type of ASC on which treatment is based | binary or continuous treatment | with or without controls | estimate |  | SE |
| total (regular, temporary, crisis) | binary | without | 0.005 |  | 0.008 |
| total (regular, temporary, crisis) | binary | with | 0.000 |  | 0.009 |
| total (regular, temporary, crisis) | continuous | without | 0.0002 | + | 0.0001 |
| total (regular, temporary, crisis) | continuous | with | 0.000 |  | 0.000 |
| crisis only | binary | without | 0.008 |  | 0.010 |
| crisis only | binary | with | 0.008 |  | 0.011 |
| crisis only | continuous | without | 0.001 | + | 0.000 |
| crisis only | continuous | with | 0.000 |  | 0.000 |

*Sources: 1VOP, COA.*

+ p<0.10; * p<0.05; (two-tailed test).

Notes: N_individuals_ = 19,091; N_observations_ = 38,182; controls: gender, age, proportion non-western minorities neighbourhood, economic deprivation neighbourhood.
