# Supplementary material for: Exposure to asylum seekers and changing support for the radical right: A natural experiment in the Netherlands
Source: PLoS One. 2021 Feb 2;16(2):e0245644. doi: 10.1371/journal.pone.0245644 (PMC7853446; doi:10.1371/journal.pone.0245644)
Supplement: Attachment — Submitted filename: reply_v01122020.docx [file pone.0245644.s003.docx]

**Letter of explanation**

We would like to thank all reviewers for their comments and for their time invested in thoroughly reading our manuscript. It is nice to see our manuscript is appreciated, valued and sparks a scientific debate.

In our opinion, the paper became stronger after incorporating reviewers’ comments and suggestions.

We made a replication package (website) for this paper (including datasets, scripts, additional tests). This website is currently hosted from the GitHub repository of the first author. We send the link of the replication website to the editor for inspection. Naturally, the Editor/PlosOne is free to share the link with the reviewers even if this would mean anonymity can no longer be ensured. We hope this replication package will contribute to the scientific debate on the impact of inflow of asylum seekers on support for the radical right and on different modelling strategies when faced with a ‘natural experiment’, a continuous ‘treatment variable’, a dichotomous outcome variable and individual-level panel data.

**Reviewer #1:** Title: Exposure to Asylum Seekers and Changing Support for the Radical Right

This paper tests how the sudden inflow of asylum seekers to a region changes voters’ support for the radical right in the region. Unlike many other studies on the support for the radical right, this paper utilizes an individual-level panel dataset on the regional level, employs a natural experiment method, and distinguishes between different types of refugee centers. Though authors’ finding that the inflow of asylum seekers strengthens the support for the radical right is not surprising, their methodological approaches contribute to the literature. Though I support the publication of this manuscript, there are some questions that should be answered beforehand.

**1.** The structure of the hypotheses is odd. Hypothesis 1 describes the positive correlation between refugee inflow and support for the PVV. Then, the two hypotheses (2a and 2b) suggest two different causal mechanisms between the two: one for the positive correlation (threat) and the other for the negative correlation (contact). I do not deny that the inflow of asylum seekers, or immigrants in general, can have dual effects (both positive and negative) through threat and contact mechanisms either on the support for the radical right or on public opinion on immigrants. Nonetheless, I think authors need to re-frame their hypotheses so that they incorporate all of these possible mechanisms and patterns.

*Following the suggestion of R#1 we reframed our hypotheses. We hope to have made more clear that the threat mechanism is likely to mediate the expected positive relation and that the positive contact mechanism is likely to supress the expected positive relation. Thus, we expect a positive relation between the inflow asylum seekers and support for the PVV (Hypothesis 1) and this is in part explained by the threat mechanism (Hypothesis 2). Recognizing that the inflow of asylum seekers can have dual effects, we expect that increased local positive interethnic contact experiences as a result of the inflow of asylum seekers can supress the positive relation between the inflow of asylum seekers and increased support for the PVV (Hypothesis 3). (see section ‘Theoretical expectations’)*

**2.** Related to the first comment, authors find and conclude that their results support H1, but not H2a and H2b. That is, though they find a positive correlation between refugee inflow and PVV support, the causal mechanisms they hypothesized are not supported by the results. Then, the question is: WHY does the refugee inflow strengthen support for the radical right? Authors need to discuss this.

*The reviewer is right that the threat (and positive contact) mechanism does not explain* regional *variation in changing support in PVV support. The question is “Why, at the local level, refugee inflow strengthen support for the radical right?”. In the revised manuscript we expanded the original discussion. An inflow of asylum seekers into the neighbourhood may not necessarily change the valence or strength of anti-immigration attitudes but their salience. We also mention the negative contact mechanism: the inflow of asylum seekers may have increased negative intergroup contact within the locality and, consequently, support for the PVV. Unfortunately, measures for issue salience and negative contact were not present in our dataset. A possible reason may also be our less ideal threat measure. See also point 3 below. (see page 18 of revised manuscript)*

**3.** Though authors distinguish economic threat and cultural threat in their theory section, their threat variable doesn’t. I understand that authors were not able to change the survey question, but they need to provide more discussion on the question wording itself. When the question was delivered to respondents, did it imply economic threat, cultural threat, or both of them to the people?

*The reviewer is right; our threat measure does not explicitly tap into economic, cultural or safety threat. The wording of our threat measure was: “I sometimes worry about the fact that my neighbourhood deteriorates because of the arrival of ethnic minorities.” We assume that when respondents evaluate the possible deterioration of their neighbourhood they will consider possible economic, cultural and safety issues. Following the suggestion of the reviewer we address the limitation of our threat measure in the revised discussion section. [see page 19]*

*That being said, in the Social and Cultural Developments in the Netherlands survey of 2018 (*[*https://doi.org/10.17026/dans-2dz-9wvy*](https://doi.org/10.17026/dans-2dz-9wvy)*), the same neighbourhood threat item was part of a larger set of threat items tapping into economic and cultural threat. In this dataset among 828 native Dutch respondents, the neighbourhood threat item correlates positively with cultural and economic threat items: ‘The coming of ethnic minorities to the Netherlands is a threat to our own culture’ (r* *= .659); ‘I sometimes am afraid that my financial prospects will decline due to the presence of ethnic minorities’ (r=.649). We think this warrants our assumption that our neighbourhood threat item will also pick up, to some extent, feelings of economic and cultural threat. However, we think it is beyond the scope of the present contribution to incorporate a rigorous discussion of item validity by employing a different dataset.*

**4.** As authors acknowledge, one critical weakness of their data is a plausible self-selection bias because respondents basically voluntarily sign up for the survey. One question related to this is: is there possibility that PVV supporters, after the sudden inflow of asylum seekers, are more motivated to accept the invitation to the survey in the 2nd wave because of, for example, their anger from the inflow? If this is true, then the self-selection bias problem occurred and it could make their results biased toward their findings. So, is there any way to make sure that PVV supporters and non-supporters had the same propensity to sign up for the survey, both in the 1st and the 2nd wave?

*Following the suggestion of reviewer#1, we investigated the possibility that PVV supporters in wave 1 are more motivated to accept the invitation to the 2nd survey than non-PVV supporters. We would like to point out that if this type of selection occurred, our results will be biased* ***against*** *our findings, because we are then less likely to observe an increase in PVV support between wave 1 and wave 2 (because of an inflow of asylum seekers) due to a ceiling effect. We indeed observe some significant selectivity; PVV supporters of wave 1 have a .79 probability to participate in wave 2, non-PVV supporters a .76 probability. This selection thus leads to conservative tests of our hypotheses. [see page 9]*

*Whether respondents of wave 1 experienced an increase in exposure to asylum seekers did not predict participation in wave 2, neither did an interaction between PVV support in wave 1 and increase in exposure to asylum seekers. Thus experiencing a ‘treatment’ is not related to selective participation. Receiving a treatment is also not related to pre-crisis PVV support.*

**5**. Authors describe three different types of refugee centers on page 11, but I don’t believe that they explained what a temporary ASC is. (Does the “crisis ASCs” on line 320 actually mean temporary ASCs?)

*We explain the three different types of refugee centers in paragraph ‘Central Agency for the Reception of Asylum Seekers’. Page 9, line 259 and further. We did mean ‘crisis ASC’ on line 320.*

*Regular reception centres (‘reguliere opvang’): long-term (minimum lease contract 2 years). Most regular reception centres already existed before the 2015 refugee crisis. Temporary centres (‘noodopvang’): mid-term (buildings are generally leased for a period of six to twelve months). Crisis centres (‘crisisnoodopvang’): short-term (no minimum lease period). The same refugees are housed in crisis centres for a maximum of 72 hours.*

**Reviewer #2:** This is an interesting paper on how the sudden inflow of refugees influenced vote intentions in the Netherlands. The strength of the paper is the possibly exogenous exposure to refugees due to the rapid inflow, combined with individual level panel data. The authors find that exposure to refugees increased the vote intention for PVV, the anti-immigration party in the study.

**1.** My main issue with the paper is how they analyze the data. The current analysis makes me not convinced that the authors estimate the effects of exposure.

The first issue regards exogeneity. The authors have a clear ambition to estimate causal effects, but is not sufficiently clear on what variation in exposure to refugees that is exogenous. Clearly, between-unit variation in exposure is not exogenous, but part of the variation between the waves might be exogenous. To me, the most promising source of exogenous variation stems from the crisis ASC, and the paper and the analysis should be centered on that source of variation.

*We fully agree with R#2 that the within-unit variation over time in exposure to asylum seekers is most interesting. This is exactly why we estimate FE models and why we focus on the within-effects when we discuss the results of our hybrid model. We argue that it is (very) likely that the within-unit variation over time in exposure to asylum seekers is to a large extent exogenous because: (1) respondents did not have time to move between the survey waves; (2) asylum seekers could not decide where they were housed; (3) the Dutch government and COA did not have time to select neighbourhoods for new temporary centres nor have time to select crisis centres (4) experiencing an inflow of asylum seekers was not related to pre-crises PVV support.*

*We fully admit that even if we assume that there was no active selection by respondents, asylum seekers or institutions of the neighbourhoods in which asylum seekers could be housed, this not fully guarantees that all variation in changing exposure to asylum seekers is exogenous. But because we use individual-level panel data we already control for time-stable unobserved heterogeneity (either by estimating a FE model or by including relevant time-stable variables into our hybrid model). This only leaves us with possible unobserved time-varying heterogeneity in exposure. Although, PVV supporters in wave 1 were more inclined to participate in wave 2 than non-PVV supporters (see R#1 comment 4) experiencing an increase in exposure of asylum seekers was not related to participation in wave 2 (not for PVV-supporters and not for non-PVV supporters). This makes one source of unobserved time-varying heterogeneity (different time trends in voting intentions between respondents who experienced an increase in exposure to asylum seekers) less likely but not impossible.*

*We also agree with R#2 that whether or not a change in exposure to asylum seekers may be assumed to be exogenous may depend on the type of ASC. We are, however, unsure for which type it is most likely to be exogenous. Our guess would be this will refer to changes in asylum seekers housed in the regular ASC which did not yet reach full capacity before the 2015 refugee crisis. R#2 would guess exogenous changes will be most likely associated with crisis centres, probably because these locations were already marked out by local governments to house citizens in times of incidents or disasters. We therefore show the impact of total exposure, and the impact of exposure to asylum seekers for each type of ASC separately.*

*We discuss the issue of the extent to which variation in exposure to refugees can be assumed to be exogenous in several places in the manuscript. We also come back to this issue in the discussion.*

*We have the ambition to estimate a model by which it is most likely that estimates refer to causal mechanisms. We stress in our manuscript that thanks to our data and modelling strategy it is more likely that our estimates refer to causal mechanism than related research in which no individual-level panel data is used and in which selection effects are more likely. We do not claim to have estimated causal mechanism and we try to refrain from causal language in our manuscript. We use sentences like “An influx in asylum seekers is related to a change in support of the PVV.” to describe our results. See also page 19 of our revised ‘conclusion and discussion’ paragraph:*

*“*

We tested our hypotheses employing individual-level panel data allowing us to control for (time-stable) unobserved heterogeneity. Given the short time-window between our survey waves, selective residential mobility did not plague our study. Moreover, exposure to asylum seekers was to a large extent random and our study therefore resembled to some extent a natural experiment. Because of these three reasons, combined with the fairly consistent results over different modelling strategies, many data and model requirements are met to give a causal interpretation to our finding that an inflow of asylum seekers into the neighbourhood is related to an increase in radical right support in this neighbourhood. However, we need to acknowledge that a natural experiment is not a true experiment and that our estimates only reached the boundary of the conventional significance criteria.

*”*

*We hope that R#2 agrees with us that our estimates are more likely to represent true causal mechanisms as compared to related previous research (see also reaction to comment 2 below).*

**2.** This leads me to the second issue which regards the analysis. I think the authors should estimate a standard differences-in-differences model using the crisis ASC as the treatment indicator (equal to one if a crisis ASC was set up in the neighbourhood between wave 1 and 2) and the wave as the post-treatment indicator. The DD estimate from this analysis might be given a causal interpretation.

*Reviewer 2 recommends us to "estimate a standard differences-in-differences model using the crisis ASC as the treatment indicator (equal to one if a crisis ASC was set up in the neighbourhood between wave 1 and 2) and the wave as the post-treatment indicator".*

*The traditional DiD model for individual-level panel data with additional time-constant covariates c_i_ is:*

*Y_it_= β_1_Time_t_ + β_2_Treat_i_ + δ(Time_t_⋅Treat_i_) + c_i_ + ϵ_it_	(1)*

*with δ being the DiD estimator and Treat_i_ the dichotomous treatment variable.*

*Formula (1) is equivalent to:*

*ΔY_i_=β_1_+δ Treat_i_ + ϵ_i_	(2)*

*Since our outcome is a binary variable there is no standard DiD model. We therefore estimated:*

*logit(Pr(ΔY_i_ =1|Treat_i_))= β_1_ + δ Treat_i_ ,	(3)*

*with ΔY_i_ =1 if the dependent outcome was 1 post-treatment (i.e. wave 2) and 0 if the dependent variable was 1 pre-treatment (wave 1). Note, that respondents who did not change support for the PVV drop out of this Fixed Effects Logistic regression analysis. Our Treat_i_ variable is the change in exposure to asylum seekers. Formula (3) is the fixed effects logistic regression model for two waves (or, more precisely, the first difference model which for two waves is equivalent to the more general fixed effects model).*

*Formula (2) demonstrates that the DiD model is a type of fixed effects model because the time constant covariates drop out of the model. With our FE-model, we are thus able to control for time-stable unobserved heterogeneity. This, together with the fact that within unit-variation in our treatment can be assumed to be to a large extent random (which we show in our manuscript), we can make strong (but not definite!) claims on causality.*

*Naturally, we are aware that because we have a binary outcome and we use a nonlinear link function we violate the common trend assumption necessary to interpret delta as the DiD estimator. Moreover, we like to point out that our original ‘treatment’ variable Treat_i_ is not a dichotomous variable, and this also makes why we cannot interpret our effect as the traditional DiD estimator. But we do not claim to estimate a DiD estimator.*

*Following the suggestion of R#2 we estimated formula (1) directly for a binary outcome variable as an additional robustness check. That is, we estimated a linear probability model (LPM), while controlling for heteroscedasticity in the error term. We did this once with our original continuous ‘treatment’ variable and once applying the dichotomization as suggested by the reviewer. For the latter model, the estimate could now be interpreted as a DiD estimator.*

*Given the comment of Reviewer#2 on post-treatment bias below we estimated the models without including our measures for contact and threat. We estimated models once with and once without the time stable covariates. The full results of all these additional DiD analyses can be found in our replication package. We summarized results with respect to the DiD estimator in Table A6. The DiD estimator based on a binary treatment variable did not reach significance but this was expected given the results presented in Table A3 in which the binary treatment variable did not reach significance either. The continuous treatment variable reached significance in the models without covariates, in line with our results based on the fixed effects logistic regressions.*

**3.** This again leads me to the third issue which is the examination of “as-if-random” exposure to refugees. The balance analysis in the paper (Table A3) is not properly explained. The appropriate way to examine balance is to conduct an F-test of whether the exogenous covariates can jointly predict the treatment. For instance, if you use the setup of crisis ASC in the respondents as the treatment you need to show that treated neighborhoods are similar to comparison neighborhoods.

*In our understanding of the literature there are quite a lot of different ways how to test for unbalance and how to take this into account. We would like to mention that we could distinguish four different ‘treatments’ (‘total inflow’, ‘inflow into regular ASCs’, ‘inflow into temporary ASCs’ and ‘inflow into crisis ASCs’) which are continuous treatment variables and not dichotomous.*

*The reviewer suggest us to conduct an F-test of whether the exogenous covariates can jointly predict the treatment. In this approach it is usually recommended to include many functions of the observed covariates (higher order terms and interactions). However, naturally, the F-test will be significant (at least for some model specifications and for some specific operationalization of our treatment variable) given our sample size and relatively few ‘treated’ respondents. We followed the strategy to reduce imbalance in our data, simply accepting that our dataset is to some extent unbalanced on key variables. Note that we provided the means of covariates for the pre- and post-matching datasets (Table A5). We observe that most difference of means between treatment and control groups decreased (and the treated and untreated groups are now of equal size). When we reduce imbalance, effects of interest become stronger (indicating some downward bias in the original dataset) and remain significant (even with increased variance in our estimates as a result of selectively pruning observations during the matching procedure).*

*Once again, we like to point out that we made a full replication package on GitHub. We have send the link to the editor, who is free to share this with the reviewers even if by this anonymity can no longer ensured. Would this manuscript be accepted for publication, it is very straightforward to replicate all tables and appendices and to try different matching strategies.*

**4.** The fourth issue is how the authors threat the contact and threat variables. To me these variables should be analyzed as outcomes that are potentially affected by exposure, they should not be analyzed as covariates (see the literature on post-treatment bias). I understand that the authors considers them as mechanisms or mediators, but one needs separate exogenous variation to properly estimate the role of mediators. Or, if the authors are willing to make strong (and in my view implausible) assumptions, they conduct a Baron-Kenny-type of mediation analysis.

*We did analyse these outcomes which may be potentially affected by increased exposure to asylum seekers. We mention in the manuscript that changes in exposure are NOT related to more threat or contact (see page 14). Hence the threat mechanism cannot mediate the impact of exposure and the positive contact mechanism cannot suppress the impact of exposure. For a (Baron-Kenny-type) analysis of mediation, the independent variable (change in exposure) should predict the mediator (change in threat and contact).*

*We understand post-treatment bias as (unnecessary) controlling in the regression model for the consequence of the treatment. However, our main results are summarized in Table 1 and Table 2. These tables summarize models in which threat and contact are not included (Models 1 and 2) and in which threat and contact are introduced in a stepwise fashion (Models 3-5).*

*In the requested DiD analysis (comment 2) we did not include our measures of threat and contact.*

*[We continued the numbering of R#2’s minor points]*

5. The attrition analysis mentioned on page 9 should be reported in the appendix.

*We followed the suggestion of the reviewer and added an appendix, Table A1.*

6. The underrepresentation of different groups (pg 9) should be presented in the appendix.

*At the request of the owners of the 1VOP dataset the descriptive statistics of these demographic variables for our total sample are not included in the main manuscript. See page 8/9: “For access to the original (anonymized) data we received from 1VOP, scholars may contact the owners of the 1VOP.” But see comment 7 below.*

7. Also, are sample weights applied?

*We added a robustness check on a weighted sample (see revised Table A3) and provided descriptive statistics on the weighted variables with respect to our FE sample (Table A5). We reach substantially similar conclusions using a weighted sample.*

**8.** The authors are probably interested in the experimental literature on contact theory, reviewed in Paluck et al. (2019, The contact hypothesis re-evaluated, Behavioural Public Policy)

*We thank reviewer for the interesting article. We refer to Paluck and colleagues’ study on page 8 of our revised manuscript.*

9. Have they considered non-linearity in the relationship between exposure and vote intention, perhaps exposure has larger effects in neighborhoods with low prior exposure (e.g. Hopkins 2010, cited in the paper)?

*Please note that our main model is already a non-linear model (logit link function) and that exposure to asylum seekers resulting from temporary and crisis centres pre-crisis is simply zero. We show in the manuscript that “The impact of exposure to refugees does not depend on pre-crisis voting intentions (cf. Karreth et al. 2015).” (see page 16). We also mention that “we tested for an interaction between initial levels of ethnic density (i.e. percentage of non-western minorities) and increases in exposure to refugees but these did not reach significance (see replication package).” (see page 16).*

**Reviewer #3:** This is a very important study. The effect to contact with asylum seekers on support for radical right-wing populist parties is an important subject to study. This is not the first study to examine this, but it is a very rigorous study. In this field, in particular given the social relevance of this research (how do values like tolerance develop, what is the societal reaction to refugees) and academic relevance in the debate between contact and threat, I think that studies like these, which rigorously look at the effect of specific events are welcome.
In particular I think this contributions stands out because of their advanced quasi-experimental design, which is apt to study the phenomenon, well-executed and convincing.

*Thank you.*

**1.** I have only very minor concerns. The first of these is that there is a typo on p.13: it now reads concerning the main effect studied in the paper “(b=0.022, se=0.12; Model 1, Table 2)” but then the effect would not be significant. This has to be (b=0.022, se=0.012) in line with the Table 2.

*We corrected the typo.*

**2.** The second is that the paper mixes the terms refugees and asylum seekers while the first term refers to people who have an official status as refugee and these second term refers to people who want that status. That means that in COA centres there only are asylum seekers and no refugees (who get their own housing once their status has been given).

*We thank reviewer#3 for pointing out this inconsistency. We changed the label refugee to asylum seeker whenever we explicitly mean the persons living in Dutch ASCs. See also page 5 where we explain our use of the terms refugee and asylum seekers.*
